# Depressed skull fracture secondary to the Mayfield three-pin skull clamp

**DOI:** 10.11604/pamj.2015.20.262.6492

**Published:** 2015-03-19

**Authors:** Salami Mohcine, El Mostarchid Brahim

**Affiliations:** 1 Department of Neurosurgery, Military Hospital of Instruction Mohammed V, Rabat, Morocco

**Keywords:** Depressed skull fracture, mayfield, diagnostic

## Image in medicine

The use of increasingly precise, intelligent neurosurgery instruments that allow intraoperative accuracy, visualization, surgical access, has led to mastery of surgical techniques and the transformation of prognosis, the Mayfield three-pin skull clamp was designed to rigidly affix a patient's head to the operating table during craniotomy drilling and delicate microneurosurgery. However, these instruments are not without risk, since several types of complications have been described. We report the case of depressed skull fracture a secondary to the Mayfield three-pin skull clamp in a patient operated for a meningioma of the posterior fossa as shown in this picture CT.

**Figure 1:**
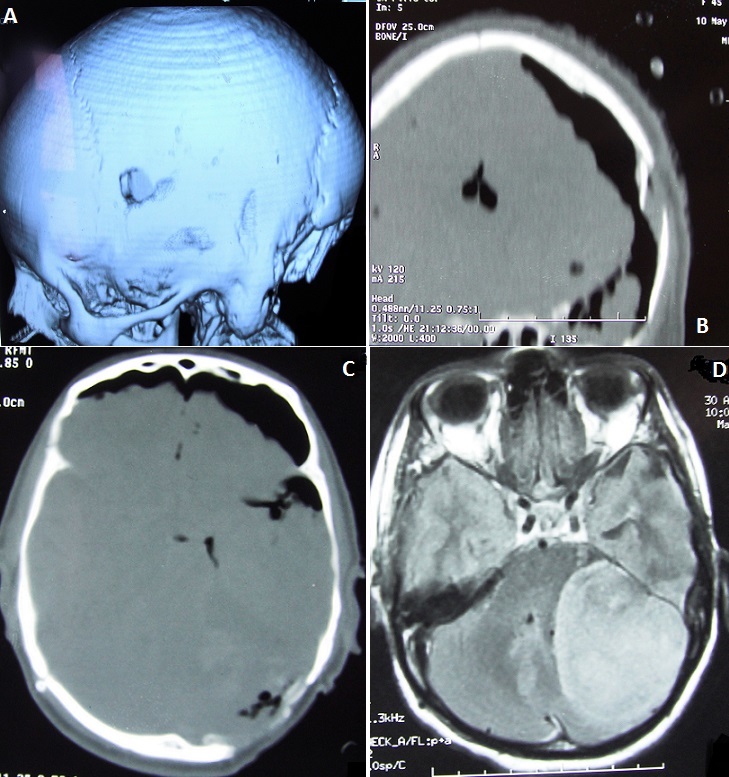
Postoperative computed tomography scan head showing left frontal depressed fracture (A, B, C) with significant pneumocephalus; (B): axial(D) T1-weighted view of magnetic resonance imaging scan demonstrate meningioma in the left posterior fossa

